# High-Resolution Ex-Vivo Imaging of Retina with a Laptop-Based Portable Endoscope

**DOI:** 10.1155/2022/1903516

**Published:** 2022-04-11

**Authors:** Yanni Ge, Kai Jin, Yao Wang, Yufeng Xu, Haitong Lu, Juan Ye

**Affiliations:** ^1^Department of Ophthalmology, The Second Affiliated Hospital of Zhejiang University, College of Medicine, Hangzhou 310009, China; ^2^SKS Health Ltd., Hangzhou 310009, China

## Abstract

**Purpose:**

Ophthalmic endoscopy is useful in vitreoretinal surgery with opaque anterior segments or anatomically challenging structures. However, standard ophthalmic endoscopy devices are usually large and costly. Thus, the development of a portable endoscope is necessary.

**Methods:**

The portable endoscope consisted of a probe, an illumination system, a high-resolution camera module, and a universal serial bus (USB). It was connected to a laptop and applied for intraocular imaging of porcine eyes in vitro. Basic parameters and pictures of the same tissue target were compared with those of the standard Endo Optiks E4 system.

**Results:**

The retinal images were displayed on the laptop screen, which clearly showed the overall appearance of the central and peripheral retina, and the details of the retinal vasculature and ciliary body. Compared with a standard endoscope, our portable endoscope is smaller and more affordable. It can be taken anywhere for intraocular imaging and vitreoretinal surgery.

**Conclusion:**

A laptop-based portable endoscope is a promising device in vitreoretinal surgery. It provides high-resolution images of intraocular tissues that should make a noticeable difference in intraocular surgery with disordered anterior segments. With its portability and high-resolution imaging, it may promote the application of endoscopes in ophthalmology.

## 1. Introduction

Endoscopy is a useful adjunct to surgical microscope ophthalmoscopy [1, 2] because it can provide non-stereoscopic, ultrahigh-resolution, intraocular images on a video monitor. Surgical microscopes fail to present details of vitreoretinal tissues with disordered anterior segments or optical aberrations. However, endoscopes overcome these limitations. The magnification and resolution of images can be increased by moving the intraocular probe closer to the target tissue. Endoscopes can also access anatomically challenging structures in the eye.

As early as 1934, endoscopy was applied for retrieving an intraocular foreign body [3]. With the continuous progress and development of endoscopic imaging, the advantages and importance of ophthalmic endoscopy have become increasingly obvious. It has been applied for the diagnosis and treatment of various intraocular diseases [4–10]. It is advantageous for patients scheduled for keratoprosthesis surgery with opaque anterior segments, following ocular trauma or endophthalmitis [11, 12].

Nowadays, the most widely available ophthalmic endoscope consists of an endo probe console and a video monitor. The console provides a high-resolution camera, xenon-light-based illumination, interfaces for fiberoptic cables, video output, and a treatment laser if needed. [13] Although this design is very practical, its bulky base unit and high cost, just like the original computer, limit its practicality. Sometimes, the image resolution cannot meet the surgeon's needs. Guilherme et al. reported their modifications which resulted in a much cheaper and portable endoscope. They removed the widefield lens of a GoPro HERO5 camera and refitted it with a C-mount and endoscopic probe, [14] but it is just a derivative of the standard endoscope. Toshio et al. developed a “proximity endoscope”. Its working distance was 3 mm, shorter than the standard endoscope; thus, it could observe the structure under the proliferative membrane much more clearly [15].

Here, we present a high-resolution endoscope, which is more portable and affordable. It can be applied for vitreoretinal surgery in rural areas.

## 2. Materials and Methods

### 2.1. Simulation Experiments

**Figure 1:**
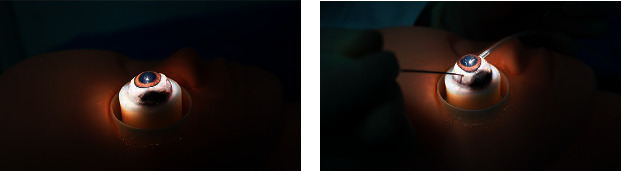
Steps of the vitreoretinal surgery. (a) Fix the porcine eye at the eye seat model. (b) Two incisions were made at the location of 4 o'clock and 10 o'clock, 2 millimeters away from the corneal limbus, with a 25-gauge needle and the probe of endoscope entering the eye.

We used ex-vivo porcine eyes for intraocular imaging since they are similar to hominin eyes. Fresh porcine eyes were enucleated at a local abattoir and transported to the laboratory in normal saline at 4°C. All animal experiments were approved by the Institutional Animal Care and Use Committee of the second affiliated Hospital, School of Medicine, Zhejiang University. First, we mounted each eye on the ophthalmological eyeball holder to provide stability (Figure 1(a)) and cut the bulbar conjunctiva 2 mm from the corneal limbus with corneal scissors. We then used conjunctival forceps to lift the conjunctival margin to separate Tenon's capsule. Subsequently, two incisions were made. One was made at the 10 o'clock position with a 25 G needle to inflate the eye to the normative intraocular pressure (IOP) with normal saline. The other was at the 4 o'clock position and allowed the endoscope probe to enter the eye (Figure 1(b)). The endoscope was sterilized with ethylene oxide before use. The experienced ophthalmic specialist adjusted the angle and distance of the probe in the eye while viewing the image on the screen to achieve clear imaging of intraocular tissues. The specialist was informed in advance that images of the same structure should be captured at the same probe insertion position and the comparable working distance from the retina.

### 2.2. Image Quality Assessment

One retina specialist did the work of image acquisition via a portable endoscope and Endo Optiks E4. The characteristics and differences of these two endoscopes were masked. Thirty fundus images were captured by these two endoscopes, respectively. All these pictures were graded by three retina specialists. Before that, they agreed to the scoring rule, which mainly focused on the visibility of the optic disc and blood vessels in the retina (3-excellent, the optic disc and peripheral blood vessels are clear enough; 2-good, the optic disc is clear, but the peripheral blood vessels are not clear enough; 1-fair, the optic disc structure and blood vessels are not clear enough; and 0-poor, only the outline of optic disc and blood vessel can be seen). Any endoscope-related information was masked. The data are presented as mean ± SD. The average scores of thirty pictures were calculated. Two-tailed Student's *t*-test was applied for statistical analysis. There is statistical significance between groups when the *P*-value is less than 0.05.

## 3. Results

### 3.1. Basic Principles and Components of the Portable Endoscope

**Figure 2:**
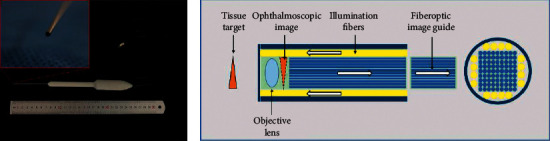
Structure of the portable endoscope. (a) Prototype overall view of the portable endoscope, with a stainless steel tube (8 centimeters in length, 19 gauge in diameter), a handle, and a USB interface. (b) Structural schematic diagram of the portable endoscope. A camera module and illumination fibers lie inside the steel tube. The illumination source is supplied from the built-in LEDs through plastic optical fibers. The camera module equipped in the handle can compose, capture, and transmit images. The images were transmitted to the PC through a fiberoptic image guide. USB: Universal Serial Bus, LED: universal serial bus.

The portable endoscope was composed of a stainless steel tube, a handle, and a universal serial bus (USB) (Figure 2(a)). The stainless steel tube was 8 centimeters (cm) in length. The distal part was the probe and was 19 gauges (*G*) in diameter. A camera module and illumination fibers were placed inside the steel tube. The camera module was equipped with an OV6948 module, which had a field of view (FOV) of 120° and the depth of field was 3–5 millimeters (mm). The SKS-SB mainboard was used. The endoscope had a built-in light emitting diode (LED) and guided light through plastic optical fibers. The power supply of the device was provided by connecting to a personal computer (PC) via USB (Supplemental Figure 1A). The light intensity of LED is 6500 lux. The temperature of the light source can be controlled below 37°C. With the equipment of electromagnetic isolation, it is safe for clinical application. The USB also allowed for image output to the PC. As shown in Figure 2(b), there was an objective lens mounted at the distal end of the probe. The light source illuminated the target tissue and formed a clear image through the objective lens. The obtained images were transmitted to the near-end video camera by the fiberoptic image guide (Figure 2(b)). Finally, the camera transmitted the video information to the PC monitor.

### 3.2. Image Quality of Two Ophthalmic Endoscopes

**Figure 3:**
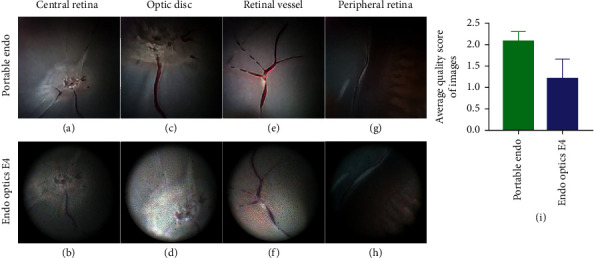
Images of retina captured by the portable endoscope and endo optics E4. An endoscopic image of the central retina with the portable endoscope (a) and E4 (b). The optic disc captured with the portable endoscope (c) and E4 (d). The retina vessels can be visualized by the portable endoscope (e) and E4 (f). The image of the ciliary body and peripheral retina captured by the portable endoscope (g) and E4 (h). The average quality score of fundus images from portable endoscope and E4 were qualified and analyzed (i)*P*=0.0420.

The porcine eyes were placed on the eyeball holder. After inflating the eye with normal saline, we inserted the probe of the portable endoscope through the second incision. The images of different retina sections from different imaging angles are shown in Figure 3. These pictures were captured with a portable endoscope or with an Endo Optiks E4 (Clinico Corp, Taiwan, China). Endo Optiks E4 is one of the most commonly used endoscopes in clinical ophthalmology, so we wanted to compare its imaging capability with that of our original portable design. First, we captured an image of the entire central retina and visualized the overall profile of the optic disc. Since the porcine eyes were ex-vivo, the retina appeared pale due to a lack of blood supply. The central retinal artery and vein containing residual blood could be seen passing through the fovea. The target tissue mentioned above could be visualized with both the portable endoscope (Figure 3(a)) and the Endo Optiks E4 (Figure 3(b)). As the probe approached the optic disc, some details of the target tissue were captured (see video, Supplementary File–Video 1). The boundary of the optic disc was distinct as captured by the portable endoscope (Figure 3(c)), while it was a little blurred in the standard endoscope image (Figure 3(d), Supplementary File–Video 3). In addition, the distribution and trend of retinal vessels were captured by the portable endoscope. Some of the retinal vasculature and branches of these vessels were visualized (Figures 3(e) and 3(f)). The average image quality score with the portable endoscope was 2.08 ± 0.13, higher than that (2.08 ± 0.13) of Endo Optiks E4 (Figure 3(i), *P* = 0.0420). Thus, the image quality of the portable endoscope proved to be good.

Due to the anatomical structure of the eye, the peripheral retina could hardly be seen by surgical ophthalmic microscopy. Thus, we switched to endoscopes. By adjusting the angle of the probe in the eye, we were able to focus the light source on the peripheral retina and monitor the imaging conditions as the probe approached the peripheral retina (see video, Supplementary File–Video 2, 4). When the tip of the probe was close to the peripheral retina, the ora serrata and ciliary body could be seen (Figures 3(g) and 3(h)). Generally speaking, the portable endoscope images were consistently better than those of the standard one.

## 4. Discussion

We designed a novel portable endoscope that not only successfully performs the functions of a traditional endoscope but also offers specific advantages in terms of portability and high-quality imaging. The portable endoscope is small and quite convenient to carry. It can be put in a special instrument box and taken wherever it is needed. Moreover, it is easy to operate. The surgeons only need to connect the portable endoscope to a PC or tablet via USB, and the portable endoscope light can be powered by the connected device's battery. Once the device's camera is turned on, the image of the target tissue can be captured and transmitted by the camera module, while simultaneously being displayed on the screen.

Endo Optiks E4 is one of the most commonly used endoscopes in ophthalmology. Therefore, we compared some of the basic parameters of the portable endoscope with those of the Endo Optiks E4 (Table 1). The highest resolution of the Endo Optiks E4 is 17,000 pixels; the resolution of the portable endoscope is 40,000 pixels, which produces much clearer images of the optic disc and retina vessels (Figure 3). Even some details of the peripheral retina could be visualized. Although the portable endoscope has nonadjustable focus, the depth of field (DOF) of the portable endoscope is 3–50 mm, so regardless of the distance between the probe and the intraocular target tissue, the image on the screen is clear. Toshio et al. developed a proximity endoscope with a working distance of 3 mm, shorter than the standard working distance of 5 mm. It offered a clear and magnified vision when it was in close approximation to the retina, but a blurred vision from a distance. [15] As is well known, ophthalmologists need a training period before they can successfully handle non-stereoscopic endoscopy in vitreoretinal surgery. High-quality images are critical in keeping ophthalmologists oriented and helping them quickly judge the tissue distance in a 2-dimensional (2-D) intraocular field. Although stereoscopic endoscopy has developed in recent years [16, 17], there are still many problems and technical challenges. [18, 19] Hence, high-resolution microscopy images remain critical in vitreoretinal surgery. We have been devoted to develop a high-definition ophthalmic endoscope. In the future, we will develop a higher-resolution endoscope to capture more details in the eye, which means we have to overcome the difficulty of fiberoptic endoscopes' relatively low pixel within the existing size. It is one of the technical challenges that we have to overcome.

The dimensions of our portable endoscope depended on the dimensions of the PC (Supplementary Figure 1). The PC we used was 32.5 × 21.6 × 17 cm^3^, which is much smaller than the 164 × 57 × 57 cm^3^ Endo Optiks E4. However, the handpiece of the portable endoscope is bulky. It is mainly due to the size of the mainboard. We will replace the existing mainboard with a smaller one in the near future, making the handle much handier. It is another technical difficulty that we have to overcome. Additionally, the cost of the Endo Optiks E4 was over US$ 30,000, equivalent to the cost of approximately 6 portable endoscopes (Table 1). The portability and affordability of the portable endoscope make it an appropriate diagnosis and treatment tool for central hospitals, as well as for emergency departments and rural areas. Scientists are keen to make endoscopes smaller and smaller. As mentioned above, Guilherme et al. reported a modified endoscope that combined the GoPro HERO5 camera with an endoscopic 23-gauge probe. [14] Because smartphones are ubiquitous, a smartphone-based laryngoscope was invented, which connects the endoscope to a smartphone with an adaptor. [20] Even tethered capsule endomicroscopy has been reported. The swallowed capsule continuously obtained 10-*μ*m-resolution cross-sectional images as they traversed the esophagus. [21] We previously developed a handheld nonmydriatic fundus camera. [22] Since most endoscope devices are large and expensive, many patients in underdeveloped countries or rural regions are not able to benefit from the related high-quality medical infrastructures. Therefore, this kind of portable and low-cost endoscope is the trend. The portability of the endoscope even may open the door to ophthalmological telemedicine. Objectively speaking, there are some limitations of this portable endoscope. The portable endoscope has a built-in LED, while the Endo Optiks E4 has an adjustable external light source for illumination. Although the light intensity of the portable endoscope is safe for clinical use, an adjustable intensity of illumination must be more humane and environmentally friendly. Besides, the 19 G probe may limit its application in clinical practice, compared with the 25 G probe of Endo Optiks E4. We will optimize the probe diameter and illumination in the near future.

## 5. Conclusion

This study introduced a novel portable endoscope. With its relatively low cost, high-resolution, convenience, and portability, it may be more useful for intraocular diagnosis and vitreoretinal treatment with blurred anterior segments, and it can be used in conditions of limited medical resources.

## Figures and Tables

**Table 1:** Comparison of the ophthalmic endoscopes.

	Endo optics E4	Portable endoscope
Dimension	164 × 57 × 57 cm	32.5 × 21.6 × 17 cm
Handpiece length	5 cm	18 cm
Portability	Restricted	Portable
Probe diameter	19 G, 23 G, 25 G	19 G
Focus	Adjustable	Non-adjustable
Angle of view	110°–140°	120°
Resolution	6,000–17,000 pixels	40,000 pixels
Light source	Xenon (external)	LED (built-in)
	Adjustable	Non-adjustable
Video output	Separate-video	USB
Cost	Over US$ 30,000	US$ 500

## Data Availability

The data used to support the findings of this study are included within the supplementary information files.

## Supplementary Materials

Supplementary Materials Supplemental Figure 1 shows the Outlook of the portable endoscope and Endo optics E4 system. Supplementary File Video 1, https://drive.google.com/file/d/1EnNaRecYCTPxjn6jFzEZ_502y3xI_AUP/view?usp=sharing, shows the imaging of optic disc by the portable endoscope. Supplementary File Video 3, https://drive.google.com/file/d/1FxPwTZcY4sxmuR1WGcs6YBsKiRvmKtGl/view?usp=sharing, shows the imaging of optic disc by the standard endoscope. Supplementary File Videos 2 and 4, https://drive.google.com/file/d/12TmrYADa5b2kycUUEMa4S4M3vAbuGXMz/view?usp=sharing, https://drive.google.com/file/d/1SWhpPqi0ml8PaQL6XZo45fnrB73FrsIr/view?usp=sharing, display the imaging of peripheral retina by the portable endoscope and the standard one.
