# Supplementary material for: High-Resolution Ex-Vivo Imaging of Retina with a Laptop-Based Portable Endoscope
Source: J Ophthalmol. 2022 Apr 11;2022:1903516. doi: 10.1155/2022/1903516 (PMC9017551; doi:10.1155/2022/1903516)
Supplement: Supplementary Materials — Supplemental Figure 1 shows the Outlook of the portable endoscope and Endo optics E4 system. Supplementary File Video 1, https://drive.google.com/file/d/1EnNaRecYCTPxjn6jFzEZ_502y3xI_AUP/view?usp=sharing, shows the imaging of optic disc by the portable endoscope. Supplementary File Video 3, https://drive.google.com/file/d/1FxPwTZcY4sxmuR1WGcs6YBsKiRvmKtGl/view?usp=sharing, shows the imaging of optic disc by the standard endoscope. Supplementary File Videos 2 and 4, https://drive.google.com/file/d/12TmrYADa5b2kycUUEMa4S4M3vAbuGXMz/view?usp=sharing, https://drive.google.com/file/d/1SWhpPqi0ml8PaQL6XZo45fnrB73FrsIr/view?usp=sharing, display the imaging of peripheral retina by the portable endoscope and the standard one. [file 1903516.f1.zip › 1903516.f1/Supp video.1903516.docx]

Supplementary File Video 1, https://drive.google.com/file/d/1EnNaRecYCTPxjn6jFzEZ_502y3xI_AUP/view?usp=sharing, which showed the imaging of optic disc by the portable endoscope.

Supplementary File Video 3, <https://drive.google.com/file/d/1FxPwTZcY4sxmuR1WGcs6YBsKiRvmKtGl/view?usp=sharing>, which showed the imaging of optic disc by the standard endoscope.

Supplementary File Video 2 and 4, <https://drive.google.com/file/d/12TmrYADa5b2kycUUEMa4S4M3vAbuGXMz/view?usp=sharing>, https://drive.google.com/file/d/1SWhpPqi0ml8PaQL6XZo45fnrB73FrsIr/view?usp=sharing, which displayed the imaging of peripheral retina by the portable endoscope and the standard one.
