# Supplementary material for: High-Resolution Ex-Vivo Imaging of Retina with a Laptop-Based Portable Endoscope
Source: J Ophthalmol. 2022 Apr 11;2022:1903516. doi: 10.1155/2022/1903516 (PMC9017551; doi:10.1155/2022/1903516)
Supplement: Supplementary Materials — Supplemental Figure 1 shows the Outlook of the portable endoscope and Endo optics E4 system. Supplementary File Video 1, https://drive.google.com/file/d/1EnNaRecYCTPxjn6jFzEZ_502y3xI_AUP/view?usp=sharing, shows the imaging of optic disc by the portable endoscope. Supplementary File Video 3, https://drive.google.com/file/d/1FxPwTZcY4sxmuR1WGcs6YBsKiRvmKtGl/view?usp=sharing, shows the imaging of optic disc by the standard endoscope. Supplementary File Videos 2 and 4, https://drive.google.com/file/d/12TmrYADa5b2kycUUEMa4S4M3vAbuGXMz/view?usp=sharing, https://drive.google.com/file/d/1SWhpPqi0ml8PaQL6XZo45fnrB73FrsIr/view?usp=sharing, display the imaging of peripheral retina by the portable endoscope and the standard one. [file 1903516.f1.zip › 1903516.f1/Supplemental Figure 1.pdf]

High- Resolution Ex-vivo Imaging of Retina with a Laptop-Based Portable Endoscope

Yanni Ge[a]†, Kai Jin[a]†, Yao Wang[a], Yufeng Xu[a], Haitong Lu[b]*, Juan Ye[a]*

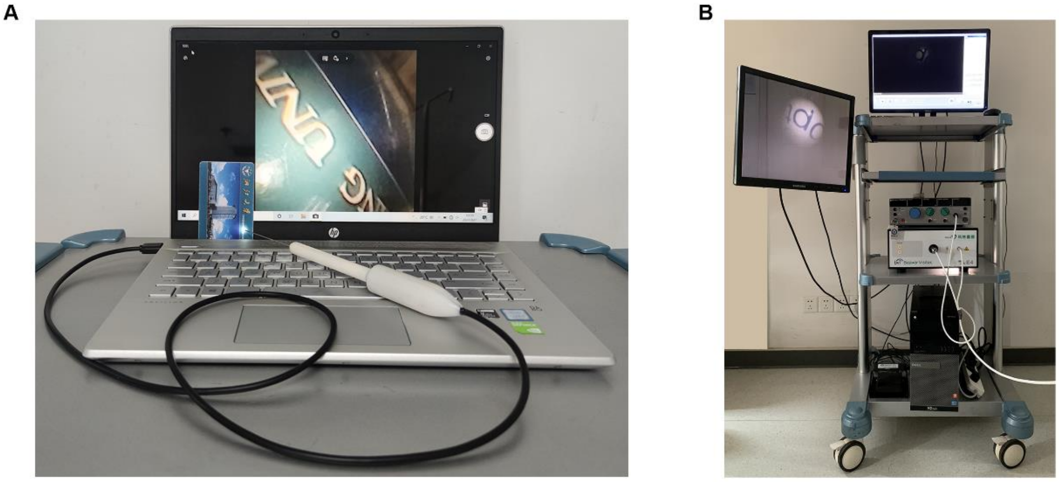

Supplemental Figure. 1. Outlook of the portable endoscope and Endo optics E4 system.

(A) The portable endoscope system consists of a probe and a handle (25 centimeters in length), and a laptop connected by USB. (B) Endo optics E4 system consists of a multifunction microendoscope and a wheeled cart housing a central console (base unit) and a two-dimensional video monitor.
